# Fast mental states decoding in mixed reality

**DOI:** 10.3389/fnbeh.2014.00415

**Published:** 2014-11-27

**Authors:** Daniele De Massari, Daniel Pacheco, Rahim Malekshahi, Alberto Betella, Paul F. M. J. Verschure, Niels Birbaumer, Andrea Caria

**Affiliations:** ^1^Institut für Medizinische Psychologie und Verhaltensneurobiologie, Universität TübingenTübingen, Germany; ^2^Fondazione Ospedale San Camillo, Istituto di Ricovero e Cura a Carattere ScientificoVenezia, Italy; ^3^SPECS - Laboratory of Synthetic Perceptive, Emotive and Cognitive Systems, Department of Technology, Center of Autonomous Systems and Neurorobotics, Universitat Pompeu FabraBarcelona, Spain; ^4^Graduate School of Neural & Behavioural Sciences, International Max Planck Research SchoolTübingen, Germany; ^5^Institució Catalana de Recerca i Estudis AvançatsBarcelona, Spain

**Keywords:** mental states decoding, EEG, mixed reality, XIM

## Abstract

The combination of Brain-Computer Interface (BCI) technology, allowing online monitoring and decoding of brain activity, with virtual and mixed reality (MR) systems may help to shape and guide implicit and explicit learning using ecological scenarios. Real-time information of ongoing brain states acquired through BCI might be exploited for controlling data presentation in virtual environments. Brain states discrimination during mixed reality experience is thus critical for adapting specific data features to contingent brain activity. In this study we recorded electroencephalographic (EEG) data while participants experienced MR scenarios implemented through the eXperience Induction Machine (XIM). The XIM is a novel framework modeling the integration of a sensing system that evaluates and measures physiological and psychological states with a number of actuators and effectors that coherently reacts to the user's actions. We then assessed continuous EEG-based discrimination of spatial navigation, reading and calculation performed in MR, using linear discriminant analysis (LDA) and support vector machine (SVM) classifiers. Dynamic single trial classification showed high accuracy of LDA and SVM classifiers in detecting multiple brain states as well as in differentiating between high and low mental workload, using a 5 s time-window shifting every 200 ms. Our results indicate overall better performance of LDA with respect to SVM and suggest applicability of our approach in a BCI-controlled MR scenario. Ultimately, successful prediction of brain states might be used to drive adaptation of data representation in order to boost information processing in MR.

## Introduction

Mixed Reality (MR) is a type of virtual reality-related technology where real and virtual worlds are merged so that real-time interaction with both physical and digital objects (Milgram, 1994; Bohil et al., 2011) is achievable. A particularly promising MR system is the eXperience Induction Machine (XIM) (Bernardet et al., 2011; Omedas et al., 2014). This technology permits to model representational elements analog to real phenomena as well as highly abstract non-representation forms describing complex high-dimensional data in a controlled environment. The exploration of data in XIM is conceptualized as an integrative narrative of varying forms where implicit and explicit responses as well as neurophysiological signals from the user can be utilized to modulate data representation (Bernardet et al., 2011; Lessiter et al., 2011; Verschure, 2011; Omedas et al., 2014).

It has been proposed that the combination of Brain-Computer Interface (BCI) technology, allowing online monitoring and decoding of mental states (Muller et al., 2008; Blankertz et al., 2010), with virtual reality systems may help to shape and guide implicit and explicit learning using ecological scenarios (Lécuyer et al., 2008; Lotte et al., 2013). Online analysis of specific brain activity has mainly been used in BCI applications for communication and control of external devices (Birbaumer and Cohen, 2007; Daly and Wolpaw, 2008), as well as for shaping behavior through neurofeedback paradigms (Delorme and Makeig, 2003; Shibata et al., 2011; Yoo et al., 2011; Caria et al., 2012; Scharnowski et al., 2012). Alternatively, the information acquired with BCI can be used to support human-computer interaction, in applications where its content is adapted to user's implicit interest, as well as for adaptive automation, affective computing, or video games (George, 2010). BCI-based real-time analysis of brain signals, with no need of participants to learn their control (sometimes referred to as “passive” BCI), can additionally be utilized to manipulate behavioral response by delivering information according to specific mental states.

Using this approach, enhancing and depressing learning and memory formation was demonstrated by triggering stimuli presentation during brain states favoring or reducing learning, which were assessed through online detection of activity in the bilateral parahippocampal areas with real-time fMRI (Yoo et al., 2011). In a similar fashion, presentation of external inputs during specific phases of neuroelectric activity might enhance or reduce participant's response.

Successful electroencephalographic (EEG)-based detection of brain states predicting participants' errors during complex cognitive decision tasks has been shown (Eichele et al., 2010). Several studies also demonstrated that brain states preceding stimulus presentation significantly affect perception (Arieli et al., 1996; Boly et al., 2007; Fox and Raichle, 2007; Fox et al., 2007; Busch et al., 2009; Mathewson et al., 2009). Building on these results, real-time information of ongoing brain states might be exploited for controlling data presentation in MR in order to boost information processing. For instance, detection of specific brain states might be used to drive changes in the level of complexity of presented information to facilitate participants' perception.

Toward this aim, we assessed to what extent multiple brain states can be discriminated during MR experience. Previous studies in the visual domain showed that real-time analysis of visual evoked potentials can detect fluctuations of perceptual dominance of each eye during binocular rivalry (Brown and Norcia, 1997). In the field of motor imagery-based BCI Millán and colleagues proposed a simple local neural classifier for the recognition of multiple mental tasks from on-line spontaneous EEG signal that achieved a recognition rate of 70% in distinguishing between relaxation, left and right hand movement imagination (Millán et al., 2002).

However, to date, clear evidence of classification of brain states during different cognitive tasks for BCI control of virtual and MR environments is still lacking. Most of studies on the integration of BCI with virtual reality focused on motor imagery, P300 and steady-state visual evoked potentials (SSVEPs) (Lotte et al., 2013). In particular, SSVEPs, permitting high information transfer rates and minimal training, seem to be suitable for BCI in virtual and MR (Martinez et al., 2007; Faller et al., 2010). Though, BCI based on continuous EEG decoding might be more flexible for monitoring brain activity during natural behavior in virtual and MR applications.

In our study, we tested classification of continuous EEG signal during spatial navigation, calculation and reading toward the implementation of BCI-controlled XIM-based MR. Spatial navigation represents a typical category of actions in virtual reality (Lécuyer et al., 2008; Lotte et al., 2013), while calculation and reading are fundamental tasks performed during information processing and data mining, and are also common cognitive processes used for mental workload assessment (Kohlmorgen et al., 2007). In particular, we performed EEG data classification using supervised classifiers based on linear discriminant analysis (LDA) and support vector machine (SVM). Furthermore, we examined predictive accuracy of our classifiers of mental workload in XIM. Increased mental workload was expected during calculation and reading as compared to spatial navigation because of larger involvement of working memory (Mayes and Koonce, 2001; Destefano, 2004; Imbo et al., 2007).

Based on previous studies showing large inter-individual differences in single-trial EEG classification of mental states in real operational environments (Kohlmorgen et al., 2007), we have used a flexible approach and calibrated our classifiers to each participant. Dynamic single trial classification was conducted using a sliding time-window shifting every 200 ms to permit applicability in a BCI-controlled MR scenario.

## Materials and methods

**Figure 1:**
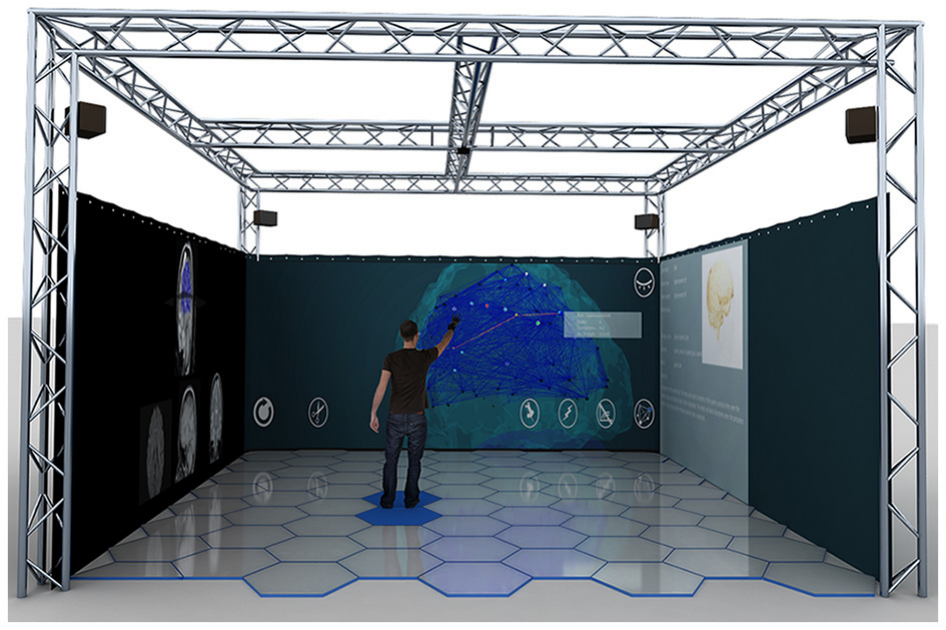
**The eXperience Induction Machine (XIM) architecture is a mixed reality integrated framework that combines a sensing system to evaluate and measure complex physiological and psychological states with a number of actuators and effectors to coherently react to the user's actions**. It is mainly constituted of an immersive room that covers a surface area of 5.5 × 5.5 m, with a height of 4 m. Eight video projectors display the scenarios into four projection screens (2.25 × 5 m) surrounding the MR room. Reprinted with permission from Betella et al. (2014).

**Figure 2:**
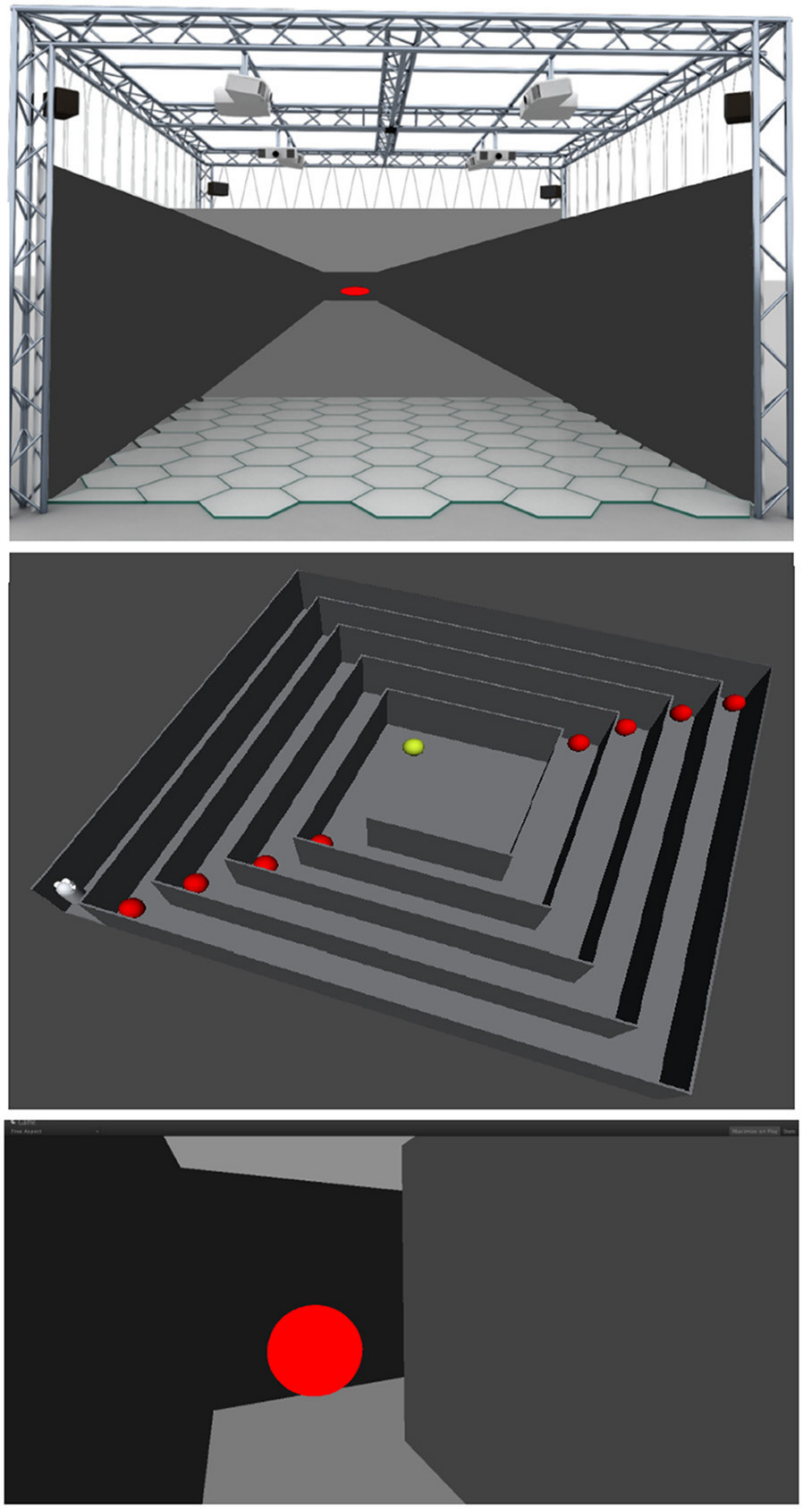
**Top:** The immersive XIM modeling the virtual maze used in the experiment (left). **Center:** View from the top of the labyrinth and the nine different targets (red spheres) that were placed in alternating corners of the path. The labyrinth size was 10 × 10 VR units (meters). Participants were required to navigate the squared spiral labyrinth until the central point (yellow sphere). Proximity to red spheres triggered the beginning of a different condition. In the first session the condition consisted of a 30 s calculation task. When the participant reached the red sphere, screen went black and a random 3-digit number was displayed in the graphical interface. The participant was asked to iteratively subtract 17 from a given number. After 30 s, the black screen faded out and the participant was asked to continue spatial navigation. In the second session, the condition consisted of a 30 s reading. The introduction of a scientific article was displayed and the participant was required to read it and press the space keyboard command when finished. **Bottom:** First person perspective of the labyrinth and a red sphere.

Five participants (29.60 ± 6.73 mean age ± *SD*, 1 female) underwent two consecutive sessions in a MR environment during which EEG signal was acquired continuously. The experiment was performed in the XIM (Bernardet et al., 2011; Omedas et al., 2014). The XIM architecture is an integrated framework that combines a sensing system to evaluate and measure complex psychological states with a number of actuators and effectors to coherently react to the user's actions (Figure 1). The internal processing of XIM is based on three main components. First, adaptive data mining that defines what data is presented to the user. Second, spatio-temporal structuring of the presented content in the form of narratives generated by the composition engines, and third an intentional, sentient agent, who controls the XIM interface and guides data exploration. XIM covers a surface area of 5.5 × 5.5 m, with a height of 4 m. Eight video projectors display the scenarios into four projection screens (2.25 × 5 m) surrounding the MR room. During each session participants experienced three different conditions, all involving the visual system: spatial navigation (SPN), reading (MER), and calculation (MEC). The user, sitting on a chair positioned in the middle of the XIM room, could navigate the virtual space by pressing the arrow keys of a keyboard. Participants were required to navigate a squared spiral labyrinth until the central point (indicated by a yellow sphere) (Figure 2). Nine different targets represented by red spheres were placed in alternating corners of the path. Proximity to red spheres triggered the beginning of a different condition. In the first session the condition consisted of a 30 s calculation task. When the participant reached the red sphere, screen went black and a random 3-digit number was displayed in the graphical interface. The participant was asked to iteratively subtract 17 from a given number. After 30 s, the black screen faded out and the participant was asked to continue spatial navigation. In the second session, the condition consisted of a 30 reading. The introduction of a scientific article was displayed and the participant was required to read it and press the space keyboard command when finished. In each session 9 SPN conditions were alternated to 8 MER or MEC conditions. An immersive 180° virtual reality application was developed and projected into the back screens of the XIM room. The VR application was developed using the Unity3D Game Engine, and adapted to fit the displays of XIM (Bernardet et al., 2011; Omedas et al., 2014). A virtual maze was modeled using Autodesk Maya (Autodesk Inc., San Rafael, CA, USA). The labyrinth size was 10 × 10 VR units (VR units permit to assign any type of units to objects' properties, e.g., weight, distance, etc., in our case they are defined as meters). The environment was constructed as an extension of the real physical space of the XIM—wall, floor, and other virtual objects were modeled so that they were perceived of real size (i.e., the point of view of the participant experiencing MR was equivalent to that of a person with average height). All participants were appropriately instructed about the experimental procedure. This study was approved by the ethics committee of the University of Tübingen.

### EEG data acquisition and preprocessing

EEG data were recorded using a 64-channels BrainAmp amplifier (Brain Products GmbH, Munich Germany). An actiCap 64-channels EEG cap (modified 10–20 system, Brain Products GmbH, Munich Germany) was used for data acquisition, referenced to the FCz, and grounded anteriorly to Fz. Only 28 surface active electrodes at the following locations were used: Fp1, Fp2, F7, F3, Fz, F4, F8, Fc5, Fc1, Fc2, Fc6, T7, C3, Cz, C4, T8, Cp5, Cp1, Cp2, Cp6, P7, P3, Pz, P4, P8, O1, Oz, O2. Electrodes impedance was reduced to 15 kΩ before data recording. EEG signals were sampled at 250 Hz.

EEG signal was first visually inspected to exclude channels affected by artifacts. Spectral analysis was then conducted on each channel to prevent our classifiers from being affected by large muscle and eye artifacts. To this aim we explored differences between SPN (low cognitive load) and MER + MEC (high cognitive load) conditions focusing on the frequencies above 20 Hz (typical of muscles artifacts) and below 6 Hz (typical of eye artifacts) (Kohlmorgen et al., 2007). The channels showing a significant difference between different workload conditions in the selected frequency bands were discarded (Kohlmorgen et al., 2007). As in Kohlmorgen et al. (2007), for each subject a customized feature selection—channel subsets, spatial filtering, frequency bands, and window lengths—was performed based on the following set of parameters: EEG channels subset {FC#, C#, P#, CP#}, {F#, FC#, C#, P#, CP#, O#}, {F#, FC#, C#, P#, CP#, O#, T7, T8}, {FC#, C#, P#, CP#, T7, T8}; spatial filter: common median reference or none; frequency band for spectral estimation: 3–15, 7–15, 10–15, 3–10 Hz; window length: 2 or 5 s. Feature extraction was performed by computing a spectral estimation within a dynamic sliding window approach shifting every 200 ms. EEG data analysis and classification were performed using MATLAB (The Mathworks, Natick, MA).

### Classification and physiological analyses

**Figure 3:**
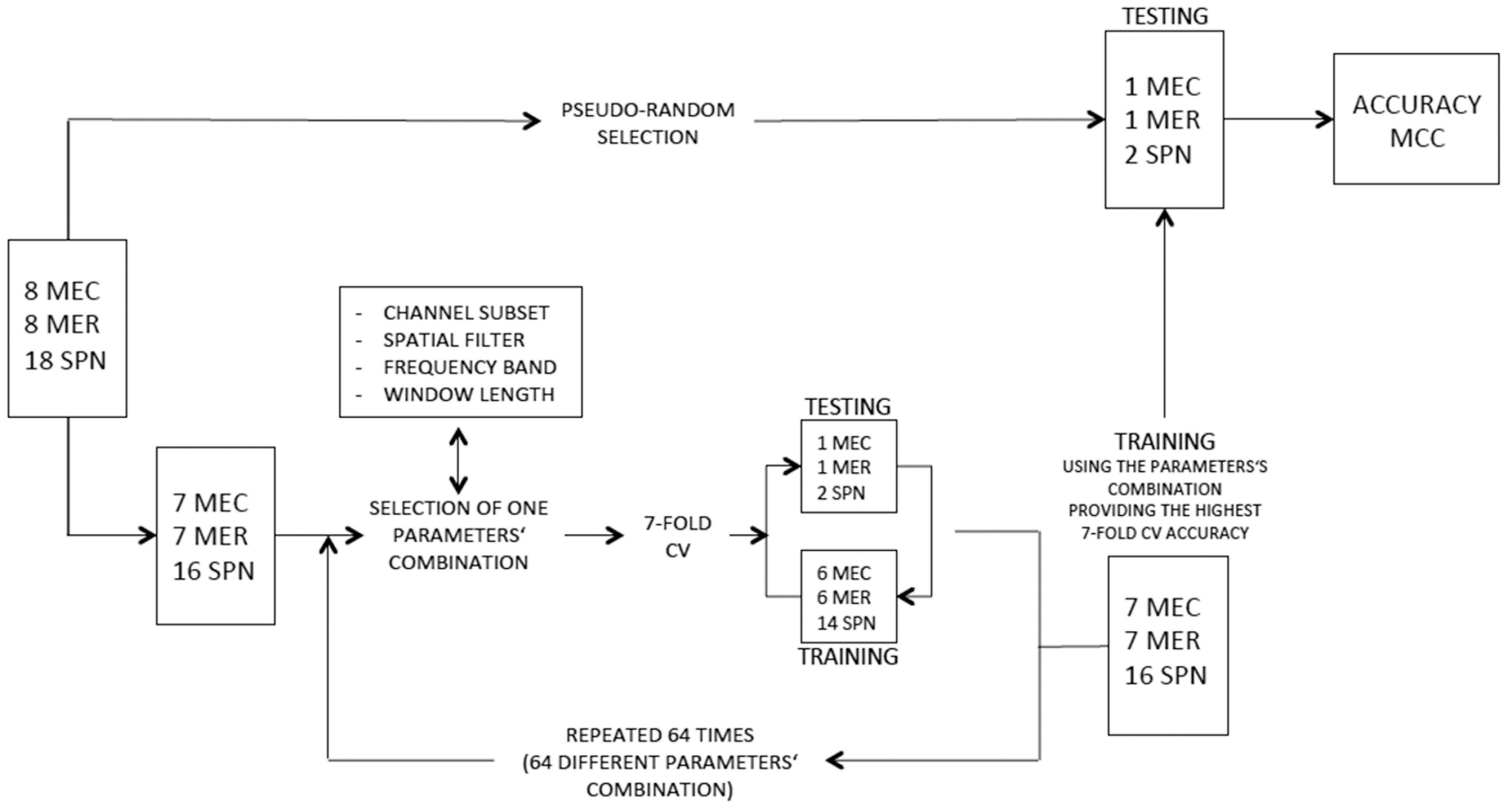
**Flow diagram depicting feature selection and estimation of classification performance for scheme 1**. A similar flow diagram was used for scheme 2 but using a different numbers of blocks and classes (HW and LW conditions).

A SVM (from LIBSVM library, http://www.csie.ntu.edu.tw/~cjlin/libsvm/faq.html#f203) with non-linear kernel (radial basis function, rbf) and a LDA classifier were tested on each subject. For SVM classification, the regularization parameter C was set to 0.6 in order to prevent over-fitting, (Cherkassky and Ma, 2004). Two different classification schemes were used. In the first scheme, the classifiers aimed to distinguish between three different classes: spatial navigation, reading and calculation. The most common decomposing strategies for multiclass SVM are the “one against one” and “one against all” binary classification approaches. The former implies the training of k^*^(k–1)/2 different binary classifiers (where k is the number of classes); each new test sample is then labeled according to the class selected by the majority of classifiers. The latter implies to build a classifier per each class to distinguish the samples of the selected class from the samples of all remaining classes; a new test sample is labeled according to the maximum outcome of all trained SVMs. A comparison of several multi-class SVM methods showed similar performance for the “one-against-all,” “one-against-one” and directed acyclic graph SVM (DAGSVM) (Hsu and Lin, 2002). However, the authors suggested that one-against-one and DAG approaches are more suitable for practical use due to reduced training time. Accordingly, we adopted the “one against one” strategy. For each subject 8 MEC, 8 MER, and 18 SPN blocks were recorded in the two sessions. A leave-one-out cross validation (CV) was used for performing feature selection and for measuring classifiers performance (see Figure 3). One MEC, one MER, and two SPN blocks were pseudo-randomly selected and retained for testing classifiers performance, while the remaining blocks were fed into a 7-fold cross-validation scheme for performing parameter selection. This procedure satisfies the requirement of independency between parameter selection samples and testing samples for classifier performance assessment. The 7-fold CV scheme was performed for each combination of parameters (i.e., channel subset, spatial filter, frequency band, and window length) to select the parameters' combination providing the best 7-fold CV accuracy (see Table 1). In each fold one MEC, one MER, and two SPN blocks were retained to compute the accuracy of the model trained on the other 6 MEC, 6 MER, and 14 SPN blocks. As duration of SPN blocks was approximately half the duration of MEC, a comparable number of samples was obtained by using for SPN double the number of MEC or MER blocks. For measuring classifiers performance the CV procedure was repeated 7 times with different training subset for each class. The 7 accuracy values were then averaged to provide the final CV accuracy.

**Table 1:** **Selected parameters in scheme 1 (top) and scheme 2 for each subject (bottom)**.

**Subjects**	**SVM**				**LDA**			
	**Channels**	**Spatial filtering**	**Window length (s)**	**Frequency band (Hz)**	**Channels**	**Spatial filtering**	**Window length (s)**	**Frequency band (Hz)**
**SCHEME 1**								
Sub 1	{F#, FC#, C#, P#, CP#, O#}	Y	5	10–15	{F#, FC#, C#, P#, CP#, O#}	N	5	3–10
Sub 2	{F#, FC#, C#, P#, CP#, O#}	Y	5	7–15	{F#, FC#, C#, P#, CP#, O#}	Y	5	3–10
Sub 3	{F#, FC#, C#, P#, CP#, O#}	Y	5	10–15	{F#, FC#, C#, P#, CP#, O#, T7, T8}	Y	5	3–10
Sub 4	{F#, FC#, C#, P#, CP#, O#}	Y	5	10–15	{F#, FC#, C#, P#, CP#, O#, T7, T8}	Y	5	3–15
Sub 5	{F#, FC#, C#, P#, CP#, O#}	N	5	10–15	{F#, FC#, C#, P#, CP#, O#, T7, T8}	Y	5	3–10
**SCHEME 2**								
Sub 1	{F#, FC#, C#, P#, CP#, O#, T7, T8}	N	5	7–15	{F#, FC#, C#, P#, CP#, O#, T7, T8}	Y	5	3–10
Sub 2	{F#, FC#, C#, P#, CP#, O#, T7, T8}	Y	5	3–10	{F#, FC#, C#, P#, CP#, O#, T7, T8}	Y	5	3–15
Sub 3	{F#, FC#, C#, P#, CP#, O#, T7, T8}	Y	5	3–10	{F#, FC#, C#, P#, CP#, O#}	N	5	3–15
Sub 4	{F#, FC#, C#, P#, CP#, O#}	Y	5	3–15	{F#, FC#, C#, P#, CP#, O#}	Y	5	10–15
Sub 5	{F#, FC#, C#, P#, CP#, O#}	Y	5	3–10	{F#, FC#, C#, P#, CP#, O#}	N	5	3–10

A second classification scheme aimed to test generalization capability of the classifier in discriminating between high (MER and MEC) and low (SPN) workload (LW and HW) independently of the type of workload. This additional scheme consisted in the application of a 11-fold CV on the merged MEC and MER datasets. The merged dataset was divided into two parts: four blocks (1 LW and 1 HW block from the MEC dataset and 1 LW and 1 HW block from the MER dataset) were pseudo-randomly selected for estimating classifier performance; the remaining 16 LW and 14 HW were fed into a 11-fold CV scheme aiming to select the best parameter setting for each subject. During each fold 14 LW and 12 HW blocks were used for training and the remaining 2 LW and 2 HW blocks for testing. The 11-fold CV scheme was performed for each combination of parameters (i.e., channel subset, spatial filter, frequency band, and window length). The parameters' combination providing the best 11-fold CV accuracy was then employed to test classifier performance on the retained four pseudo-randomly blocks (see Table 1). Features selection for the first and second classification scheme included a 5 s window length and the common median reference (in 15 out of 20 cases).

Feature selection process allowed to determine two most discriminant channels' subsets out of the four considered: {F#, FC#, C#, P#, CP#, O#} and {F#, FC#, C#, P#, CP#, O#, T7, T8} (see Table 1).

The Matthews correlation coefficient (MCC) was additionally computed, as it guarantees more robustness to performance variability in binary classification accuracy by taking into account differences in data dimensionality (Baldi et al., 2000). *MCC* ranges between -1 and 1, from total disagreement to agreement between prediction and observation, respectively, and with 0 indicating completely random prediction.

EEG spectral differences among the three conditions were inspected considering 3–7 and 8–12 Hz bands. Spectral differences between HW and LW conditions were assessed using a non-parametric Wilcoxon signed-rank test considering each of the 26 channels of all participants for 3–7 and 8–12 Hz bands separately.

## Results

**Figure 4:**
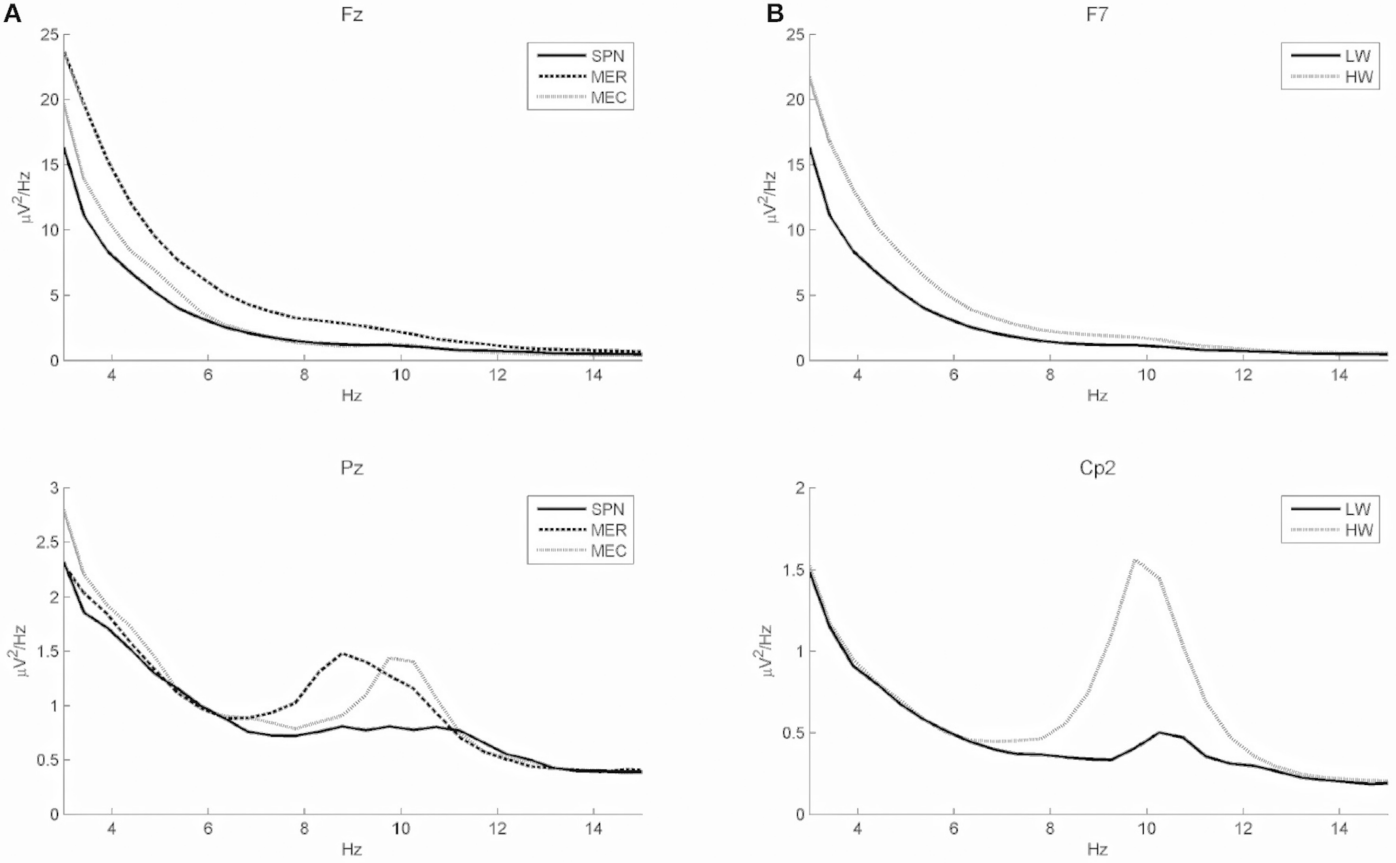
**(A)** Power spectra (grand average across all subjects) of two discriminative channels of all conditions in frontal and parietal areas (Fz and Pz). Solid, dashed, and dotted lines represent the grand average power spectrum for SPN, MER, and MEC tasks, respectively. Fz shows a clear power difference among all conditions in the 3–7 Hz band, whereas Pz shows a clear power difference in the 8–12 Hz band. **(B)** Power spectra (grand average across all subjects) of channels showing positive peaks during high (HW) as compared to low (LW) mental workload (left frontal area, F7, and central parietal region, Cp2).

**Figure 5:**
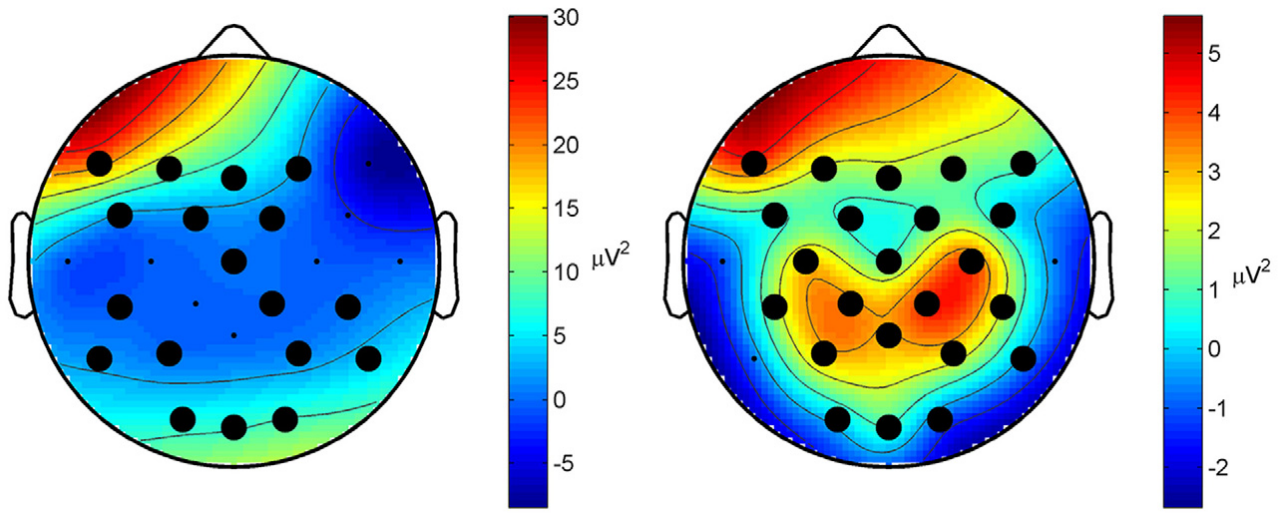
**Topographic distribution of the spectral difference in the 3–7 Hz band (left) and in the 8–12 Hz band (right) between HW and LW (grand average across all subjects)**. Larger dots indicate those channels where a significant difference was measured (*p* < 0.001). Distribution of the 8–12 Hz band power difference shows significant positive peaks in the left frontal area (F7) and bilateral parietal regions (Cp1 and Cp2). Distribution of the 3–7 Hz band power shows a significant positive peak in the frontal area (F7), and several negative peaks in central and parietal areas.

Classification performance of continuous EEG data during the three mental states SPN, MEC and MER is reported in Table 2. The LDA based classifier generated on average the highest accuracy (83.30%, *MCC* = 0.72) across all subjects, with peaks of 89.72% for accuracy and 0.84 for *MCC* in subject 2. The SVM based classifier generated on average the lower accuracy (65.68%, *MCC* = 0.45). The results of the classification between HW and LW are reported in Table 3. As for the first scheme, LDA yielded on average the highest accuracy (88.56%, *MCC* = 0.74) across all subjects, with peaks of 96.92% for accuracy and 0.92 for *MCC* in subject 1. SVM yielded on average a lower accuracy (86.59%) and a lower *MCC* value (0.63). Visual inspection of EEG power spectrum at representative discriminative channels (Fz and Pz, as they are usually less affected by muscle artifacts) showed power changes at theta frequencies (3–7 Hz) and at alpha band (8–12 Hz) among all three conditions (see Figure 4A). Moreover, increased alpha band power in frontal regions and theta band power in the frontal and parietal regions was measured during reading and calculation with respect to spatial navigation (see Figure 4B). Topographic distribution of the 8–12 Hz band power difference comparing HW to LW conditions showed positive peaks of neuroelectric activity in the left frontal area, F7, and bilateral parietal regions, Cp1 and Cp2 (*p* < 0.001, see larger dots in Figure 5, right). Topographic distribution of the 3–7 Hz band power difference comparing HW to LW conditions shows a significant positive peak in the left frontal area (F7) and several negative peaks in central and parietal areas (*p* < 0.001, see larger dots in Figure 5, left), except channels F8, Fc6, T7, C3, C4, T8, Cp1, Pz.

**Table 2:** **Results of the classification of spatial navigation, reading, and calculation**.

**Subjects**	**SVM**		**LDA**	
	**Acc (%)**	***MCC***	**Acc (%)**	***MCC***
Sub1	75.63	0.60	81.59	0.69
Sub2	54.76	0.36	89.72	0.84
Sub3	73.36	0.49	79.07	0.61
Sub4	60.35	0.36	76.56	0.62
Sub5	64.29	0.45	89.56	0.82
Average	65.68	0.45	83.30	0.72

*The accuracy, MCC, and average values for LDA and SVM classifiers are reported for each participant*.

**Table 3:** **Results of the classification between LW and HW**.

**Subjects**	**SVM**		**LDA**	
	**Acc (%)**	***MCC***	**Acc (%)**	***MCC***
Sub1	87.59	0.61	96.92	0.92
Sub2	85.43	0.63	93.72	0.86
Sub3	90.89	0.75	87.12	0.69
Sub4	80.56	0.59	73.72	0.54
Sub5	88.46	0.59	91.3	0.68
Average	86.59	0.63	88.56	0.74

*The accuracy, MCC, and average values for LDA and SVM classifiers are reported for each participant*.

## Discussion

Our study investigated brain states classification during the execution of multiple cognitive processes in MR. To this end we explored continuous EEG data decoding during performance of MR relevant tasks such as spatial navigation, calculation and reading in XIM, using LDA and SVM based classifiers. Results of our first classification scheme, aiming to discriminate spatial navigation, calculation and reading, showed high performance of both LDA and SVM classifiers, with an average accuracy of around 83% for LDA and of around 65% for SVM. Results of our second classification scheme, aiming to decode mental workload independently on the workload task, showed high classification performance of both SVM and LDA (on average both above 86%). Successful decoding of all mental states was achieved considering a 5 s time-window shifting every 200 ms, permitting online applications with a bit-rate of about 5 bits/s.

Previous EEG-BCI mainly investigated mental states decoding considering different states separately, for instance motor imagery, attention, performance capability, emotional arousal, or brain activity prefiguring behavioral errors (Millán et al., 2002; Muller et al., 2008; Schubert et al., 2009; Eichele et al., 2010). However, the capability of decoding multiple brain states is particularly important for BCI-controlled MR as it would allow the implementation of more flexible scenarios with less behavioral constraints. A previous study aiming to implement a BCI for the recognition of multiple mental tasks from on-line spontaneous EEG signal reported a recognition rate of 70% in distinguishing between relaxation, left and right hand movement imagination using a simple local neural classifier (Millán et al., 2002). In addition, the authors performed a preliminary analysis in one subject to test generalization of two local neural classifiers in discriminating between three tasks—relaxation, arithmetic subtraction, and left hand movement imagination, as well as relaxation, cube rotation and left hand movement imagination; performance accuracy reached over 90% of correct prediction on the combined task (Millán et al., 2002). A more recent EEG classification study reported successful offline multi-class discrimination of several conditions, such as resting, mental calculation, mental writing and rotation, by combining wavelet transform decomposition for feature selection and a feed-forward neural network with one-step secant algorithm (Upadhyay, 2013).

Here, we tested a simpler approach using LDA and SVM, also on the basis of the results of a comparative analysis of multi-class EEG classifiers for BCI, such as LDA, Nearest Neighbor Classifier (NNC) and linear SVM, indicating that LDA provides the highest classification accuracy with low dimensional feature space (Lee et al., 2005). In line with these results both our classification schemes showed better performance of LDA with respect to SVM.

Increased accuracy of SVM classifier would also be achievable through additional optimization procedure applied to its parameters (i.e., C and γ), typically via cross-validation, but this would result in a substantial increase of the computational time. On the other hand, SVM can achieve higher performance as compared to LDA with high dimensional feature vectors (Lee et al., 2005).

Because of large intersubject variability of EEG data, subject-dependent classifiers, as those used here, guarantee better performance than subject-independent classifiers (Lotte and Ang, 2009) but they require an initial offline calibration during which the participants need to evoke specific mental state by performing appropriate tasks (supervised classifier). However, a subject-independent BCI system with no need of training, implemented using a combination of large datasets of subject-dependent classifiers into a single subject-independent classifier, demonstrated performance similar to that of subject-dependent methods (Fazli et al., 2009). In addition, Vidaurre and colleagues investigating co-adaptive learning using machine learning techniques implemented a subject-independent supervised classifier with no need of offline calibration procedure that showed good performance even in participants that are not able to control conventional BCI (Vidaurre et al., 2010). These promising findings suggest the possibility to extend to use of subject-independent classifiers in BCI applications.

Our subject-dependent LDA-based classifier provided the highest accuracy mostly using 3–10 and 3–15 Hz frequency power bands. Spectral analysis indicated changes at theta band (3–6 Hz) as well as at alpha band (8–12 Hz) between different mental states in frontoparietal regions. Alpha band increase in the frontal area was observed for all conditions, whereas both theta and alpha bands increase in the parietal regions was larger for reading and calculation with respect to spatial navigation. Accordingly, high workload, that included both reading and calculation, compared to low workload—spatial navigation—showed significant power differences at 3–6 and 8–12 Hz bands. In particular theta band maximum was observed in the left frontal area, while alpha band peaked in the left frontal and bilateral parietal regions.

Despite intersubject variability and our small sample size, the observed alpha band increase in the bilateral parietal areas is in line with previous results reporting alpha changes in bilateral parietal and occipital brain regions associated with mental workload, task engagement or attention (Humphrey and Kramer, 1994; Pope et al., 1995; Kohlmorgen et al., 2007). The measured changes in the theta band power are also in line with previous studies indicating that theta oscillations are related to spatial navigation as well as encoding and retrieval of spatial information (Kahana et al., 1999; Bischof and Boulanger, 2003). In particular, high amplitude theta activity, mainly in the left frontal and right temporal cortices, has been measured during navigation in a virtual maze (Kahana et al., 1999). Other studies corroborated these results and showed that the frequency of theta episodes is directly associated with the difficulty of maze navigation (Bischof and Boulanger, 2003). In light of previous studies our results indicate that indeed the here adopted reading and calculation tasks required increased allocation of mental resources with respect to spatial navigation.

In addition, we observed delta frequencies (3 Hz) power changes during all conditions in frontal regions. These results are in line with previous studies indicating increased EEG oscillations in the range 1–3.5 Hz in frontal regions associated with different cognitive processes (Harmony, 2013), in particular during internal concentration and calculation (Fernandez et al., 1995).

Our mental states classifiers can equally be employed for real-time analysis of frequency bands. Online monitoring of alpha and theta bands power would be important for assessing participants' performance as these bands reflect cognitive and memory processing (Klimesch, 1999). In online MR-combined BCI applications the information stream provided to the user could be adapted to the current workload as indicated by alpha and theta oscillations. Kohlmorgen et al. (2007) proposed the use of ratios of activity in alpha (8–12 Hz) or theta (3–7 Hz) bands to compute an index of the user task engagement.

Alpha band is also critical for visual perception, in particular medium and lower amplitudes can reflect improved performance in somatosensory and visual discrimination tasks (Pfurtscheller and Lopes Da Silva, 1999; Hanslmayr et al., 2005; Palva and Palva, 2007; Van Dijk et al., 2008). Moreover, decrease in the alpha frequencies (8–12 Hz) before target onset was associated with augmented visual target detection (Ergenoglu et al., 2004). Further studies confirmed this observation by showing that the amplitude of prestimulus ~10 Hz oscillations correlated with the detection of the upcoming target: the smaller the amplitude, the more likely the target would be detected (Van Dijk et al., 2008; Busch et al., 2009; Mathewson et al., 2009). On the contrary, stronger pre-stimulus alpha frequency band amplitude has been linked to increased cognitive performance (Neubauer and Freudenthaler, 1995; Klimesch, 1999). Thus, online inspection of alpha wave oscillations might be used for triggering stimuli presentation so as to optimize stimulus detection, as well as for improving interpretation of novel information and data mining since alpha band activity has also been associated with learning (Klimesch, 1999).

In conclusion, our LDA classifier is sufficiently flexible and powerful for the implementation of a MR-combined BCI system. Successful classification of mental states based on subject-specific single trials EEG indicates the possibility to combine BCI technology with the XIM so that brain activity could drive the adaptation of data representation. A possible way to refine our BCI in MR is the use of an asynchronous modality where participants do not need to follow a fixed repetitive scheme to switch from one mental task to another one. Asynchronous BCI, allowing individuals to decide when to perform a mental task and when to stop it and switch to another one, are more flexible and adaptive to different scenarios (Millán et al., 2002; Millan Jdel and Mourino, 2003). Ultimately, this approach would permit to model the user experience in the XIM as common product between the initial data representations and the changes made interactively as consequences of users' neurophysiological signals associated with spontaneous behavior.

### Conflict of interest statement

The Review Editor Dr. Emanuele Pasqualotto declares that, despite having collaborated with some of the authors, the review process was handled objectively. The authors declare that the research was conducted in the absence of any commercial or financial relationships that could be construed as a potential conflict of interest.
